# Comparison of the Efficacy and Prognosis of Different Treatment Strategies for Spinal Tuberculosis Patients Undergoing Vertebral Augmentation Surgery by Mistake

**DOI:** 10.1111/os.70373

**Published:** 2026-07-07

**Authors:** Xiong Xu, Yatao Wang, Litao Li, Zhanpeng Luo, Ning Liu, Yunfeng Wu, Cunshuo Wang, Bo Wang, Enning Cui, Long Yu, Xu Cui

**Affiliations:** ^1^ Graduate School of Hebei North University Zhangjiakou Hebei Province China; ^2^ The Fourth Medical Center of Chinese PLA General Hospital Beijing China

**Keywords:** bone cement, spinal tuberculosis, vertebral augmentation

## Abstract

**Objective:**

Early diagnosis of spinal tuberculosis remains challenging, and inappropriate percutaneous vertebral augmentation can aggravate lesions and worsen clinical symptoms. Relevant clinical evidence on subsequent standardized management remains limited. This study aimed to investigate the clinical efficacy and prognostic outcomes of different treatment strategies in patients with spinal tuberculosis following inappropriate percutaneous vertebral augmentation, and to analyze the clinical characteristics of the patients and provide clinical data for the differential diagnosis of spinal tuberculosis.

**Methods:**

The clinical data of 53 patients with spinal tuberculosis who underwent vertebral augmentation between January 2012 and January 2024 were retrospectively analyzed. There were 26 males and 27 females, with a mean age of 70.33 ± 5.88 years (range 53–86 years). Thirty‐one patients had thoracic tuberculosis, and 22 had lumbar tuberculosis. According to the ASIA Impairment Scale, 7 patients were grade B, 15 were grade C, 24 were grade D, and 7 were grade E. At admission, 52 patients had elevated erythrocyte sedimentation rates (ESRs) and C‐reactive protein (CRP) levels, 36 had positive T‐SPOT results, and 1 patient had a normal ESR and CRP level and negative T‐SPOT result. Twenty‐seven patients had single‐vertebral involvement, and 26 had multiple‐vertebral involvement. Patients were divided into two groups, and a retrospective cohort comparative analysis was conducted: Surgical group (35 patients) received posterior spinal canal decompression, bone graft fusion, and internal fixation; conservative group (18 patients) received non‐surgical treatment. Outcomes included ESR, CRP level, VAS score, ASIA grade, and MBI score. Enumeration data were analyzed with the χ^2^ test for intergroup differences in proportions. Normally distributed continuous data were compared using the independent samples t‐test. Non‐normally distributed continuous data were analyzed using the Mann–Whitney U test. Paired comparisons were performed using the Wilcoxon signed‐rank test. Repeated measures data were analyzed using the rank‐sum test.

**Results:**

The follow‐up period ranged from 18 to 36 months. Early postoperative increases in the ESR and CRP level were significantly greater in the surgical group than in the conservative group, but inflammatory marker levels normalized within 3–6 months in both groups. Before treatment, there were no significant differences in the ESR, CRP level, VAS score, MBI, or ASIA grade between the groups (all *p* > 0.05). At 3 months and at the final follow‐up, the ESR, CRP level, and VAS score decreased significantly and the MBI and ASIA grades improved significantly in both groups (all *p* < 0.05). The MBI was significantly better in the surgical group at 3 months (*p* < 0.05). At the final follow‐up, no significant differences were found between the groups in any index (all *p* > 0.05).

**Conclusion:**

This retrospective cohort study shows that compared with conservative treatment, surgical treatment results in faster symptomatic and functional recovery in patients with neurological compression after mismanaged vertebral augmentation for spinal tuberculosis. For patients without significant neurological compression or those who are unfit for surgery, conservative treatment achieves satisfactory long‐term efficacy, although the recovery time is longer. Long‐term outcomes are comparable between the two strategies.

## Graphical abstract

Based on the clinical characteristics of patients with spinal tuberculosis after percutaneous vertebral augmentation, the corresponding diagnosis and treatment strategies were explored, and clinical data for the differential diagnosis of spinal tuberculosis were provided.

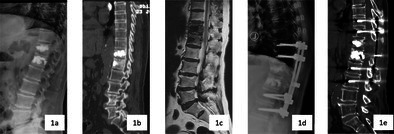

## 1 Introduction

Tuberculosis remains one of the three major infectious diseases worldwide and is a severe global public health problem [1]. According to the World Health Organization (WHO), an estimated 1.25 million people died from tuberculosis globally in 2023 [2]. Spinal tuberculosis is the most common form of extrapulmonary tuberculosis, accounting for approximately 50% of skeletal tuberculosis cases [3]. However, the early diagnosis of spinal tuberculosis remains challenging [4, 5].

Elderly patients often present with nonspecific clinical and imaging manifestations, leading to frequent misdiagnoses of vertebral compression fractures and inappropriate vertebral augmentation [6, 7]. Notably, spinal tuberculosis is an absolute contraindication to vertebral augmentation. The presence of bone cement and delayed antituberculosis treatment often exacerbate infection, accelerate disease progression, and may lead to catastrophic neurological consequences [8, 9]. With population aging and the increasing incidence of osteoporotic vertebral compression fractures, the number of patients misdiagnosed with spinal tuberculosis and undergoing inappropriate vertebral augmentation has increased annually [10].

This study aimed to achieve the following objectives: (i) to perform a retrospective analysis of the clinical characteristics of such patients; (ii) to evaluate the clinical efficacy and prognostic outcomes of different treatment strategies in these patients; (iii) to supply clinical data for the differential diagnosis of patients with spinal tuberculosis.

## 2 Materials and Methods

### 2.1 Inclusion and Exclusion Criteria

The inclusion criteria for patients were as follows: (1) patients who underwent vertebral augmentation and presented with persistent or progressive pain at the treated level, radiating pain, or systemic tuberculous symptoms, including night sweats, low‐grade fever, and weight loss; (2) a definitive diagnosis of tuberculosis confirmed by positive *Mycobacterium tuberculosis* culture, Xpert test, or histopathological examination; (3) imaging findings consistent with those of spinal tuberculosis on plain radiography, computed tomography (CT), or magnetic resonance imaging (MRI); and (4) signed informed consent.

The exclusion criteria were as follows: (1) tuberculous lesions involving vertebrae different from those treated with augmentation; (2) fractures caused by trauma, tumor, metabolic bone disease, or other spinal infections; and (3) incomplete follow‐up data, loss to follow‐up, or death during follow‐up.

### 2.2 General Data

**FIGURE 1:**
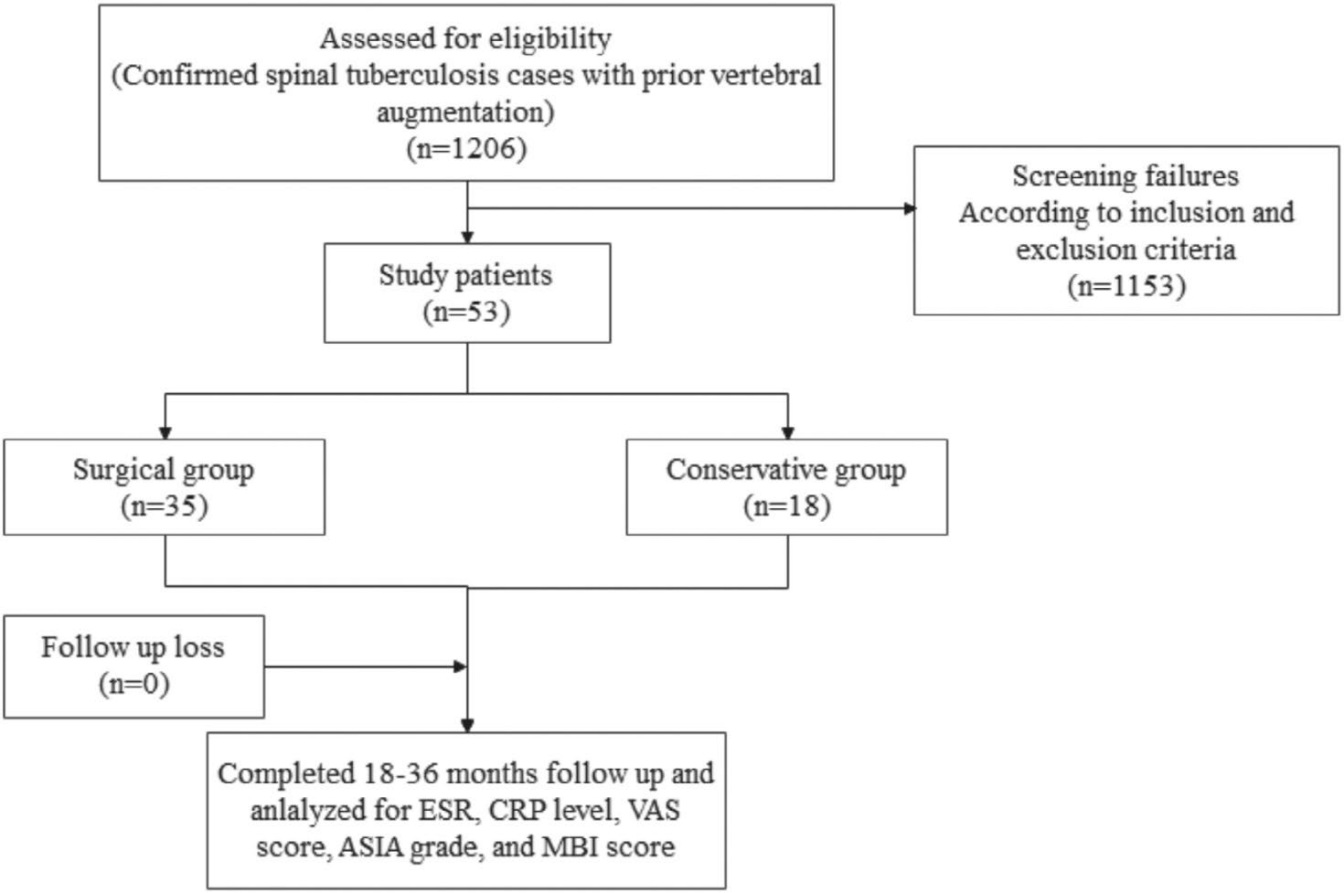
Patient enrollment flow diagram.

A total of 1206 patients with a history of vertebral augmentation and a subsequent diagnosis of spinal tuberculosis were admitted between January 2012 and January 2024. Among them, 53 patients (4.39%) met the inclusion and exclusion criteria and were included in the study. There were 26 males and 27 females, with a mean age of 70.33 ± 5.88 years (range 53–86 years). Thirty‐one patients had thoracic tuberculosis, and 22 had lumbar tuberculosis. Twenty‐seven patients underwent single‐level augmentation, and 26 underwent multi‐level augmentation. Neurological function was evaluated using the American Spinal Injury Association (ASIA) classification [11, 12, 13] (Figure 1).

Surgical group (*n* = 35) included 18 males and 17 females, with a mean age of 70.657 ± 4.491 years, who were treated with posterior decompression, debridement, bone graft fusion, and instrumentation. Conservative group (*n* = 18) included 8 males and 10 females, with a mean age of 69.722 ± 8.050 years, who were treated with non‐operative management.

All 53 patients (100%) experienced localized pain and limited mobility; 46 patients (86.79%) experienced radiating pain and muscle weakness, with a mean duration of 4.226 ± 0.462 months (range 3–6 months). Thirty‐seven patients (69.81%) presented with systemic tuberculous symptoms. The median VAS score was 8.000 (7.000, 8.000) in the surgical group and 7.500 (7.000, 8.000) in the conservative group. The median MBI score was 50.000 (45.000, 50.000) in the surgical group and 45.000 (45.000, 50.000) in the conservative group.

All patients underwent preoperative X‐ray, CT, and MRI. Paravertebral abscess was detected in 37 patients (69.81%). Fifty‐two patients (98.11%) had elevated erythrocyte sedimentation rates (ESRs) and C‐reactive protein (CRP) levels, and 36 patients (67.92%) had positive T‐SPOT results. The diagnosis was confirmed by microbiological or pathological examination. This study was approved by the Ethics Committee of the Chinese People's Liberation Army General Hospital (No. 309201204151037).

### 2.3 Perioperative Preparation

Routine preoperative evaluation included T‐SPOT, ESR, CRP levels, Brucella agglutination test, procalcitonin, interleukin‐6, routine blood test, and fungal screening. Imaging examinations were used to determine the extent of lesions, vertebral destruction severity, and degree of nerve compression. Standard anti‐tuberculosis chemotherapy recommended by WHO was administered: isoniazid 5 mg/kg (max 300 mg), rifampicin 10 mg/kg (max 600 mg), ethambutol 15–25 mg/kg, and pyrazinamide 15–30 mg/kg (max 2 g), orally once daily for a total duration of 18–24 months [14]. Comorbidities, cardiopulmonary function, and nutritional status were systematically assessed.

### 2.4 Treatment Methods

Treatment strategies were individualized based on the degree of neurological compression, progression of neurological deficits, and general condition.

Patients in the surgical group were treated with the posterior midline approach, spinal canal decompression, posterolateral bone grafting, and instrumentation.

Patients in the conservative group received standardized anti‐tuberculosis chemotherapy combined with nutritional support, medical comorbidity management, and regular monitoring.

### 2.5 Perioperative Management

Specimens obtained from patients in the surgical group were sent for pathological examination, bacterial culture, and molecular testing. Anti‐tuberculosis treatment was continued for at least 18 months and extended to 18–24 months for drug‐resistant patients.

### 2.6 Follow‐Up and Outcome Assessment

Follow‐up was performed at 1, 2, 3, 6, 9, and 12 months, then every 6 months for 1–2 years, and annually thereafter. The following was assessed at follow‐up: ① pain: visual analog scale (VAS); ② spinal fusion: Brantigan‐Steffee criteria; ③ neurological function: ASIA grade; ④ daily living ability: modified Barthel index (MBI) [15, 16]; and ⑤ lesion healing: serial imaging evaluation.

### 2.7 Statistical Analysis

All the data in this study were statistically analyzed using SPSS 26.0 software. Enumeration data (sex, T‐SPOT result, lesion segment, duration of anti‐tuberculosis medication and location) were analyzed with the *χ* ^2^ test for intergroup differences in proportions. Normally distributed continuous data (age, number of involved vertebrae, disease duration and follow‐up period) are presented as the mean ± SD and were compared using the independent samples *t*‐test. Non‐normally distributed continuous data (VAS scores, ESR, CRP Levels, ASIA grade and MBI score) are presented as medians (interquartile ranges) and were analyzed using the Mann–Whitney U test. Paired comparisons were performed using the Wilcoxon signed‐rank test. Repeated measures data were analyzed using the rank‐sum test. *p* < 0.05 was considered to indicate statistical significance.

## 3 Results

### 3.1 Baseline Comparison

Comparisons of preoperative general data between the two groups revealed no significant differences in sex distribution, age, T‐SPOT results, lesion segments (single segment/multiple segments), duration of antituberculosis drug use, lesion segment location (thoracic/lumbar), number of diseased vertebrae or disease course between the two groups (*p* > 0.05), as shown in Table 1.

**TABLE 1:** Baseline characteristics of the patients in the two groups.

Item	Surgical group	Conservative group	*χ* ^2^/*t*	*p*
Sex			0.232	0.63
Male	18 (51.429)	8 (44.444)		
Female	17 (48.571)	10 (55.556)		
Age (years)	70.657 ± 4.491	69.722 ± 8.050	−0.457	0.652
T‐SPOT result			0.231	0.631
Positive	23 (65.714)	13 (72.222)		
Negative	12 (34.286)	5 (27.778)		
Lesion segment			0.01	0.922
Single	18 (51.429)	9 (50.000)		
Multiple	17 (48.571)	9 (50.000)		
Anti‐TB drugs within 2 weeks			0.012	0.912
Yes	15 (42.857)	8 (44.444)		
No	20 (57.143)	10 (55.556)		
Location			0.077	0.781
Thoracic	20 (57.143)	11 (61.111)		
Lumbar	15 (42.857)	7 (38.889)		
No. of involved vertebrae	1.514 ± 0.507	1.500 ± 0.514	−0.097	0.923
Disease duration (months)	4.057 ± 1.259	4.556 ± 0.922	1.485	0.144
Follow‐up period (months)	23.83 ± 6.991	25.56 ± 6.543	−0.855	0.397

### 3.2 Comparison of Perioperative Conditions and VAS Scores Between the Two Groups

In the surgical group, all operations were completed successfully, and no complications, such as anesthesia accidents, cerebrospinal fluid leakage, sinus formation, or deep vein thrombosis, occurred during or after the operation. Compliance with anti‐tuberculosis drug treatment was good in both groups, and all patients completed the 18‐month standard anti‐tuberculosis drug course under standardized supervision.

Between‐group comparisons revealed that before treatment, there was no significant difference in the VAS score between the conservative group and surgical group (z=−1.887,p=0.059); the baseline scores were comparable. At the last follow‐up, there was still no significant difference in the VAS score between the two groups (z=−1.211,p=0.226).

**FIGURE 2:**
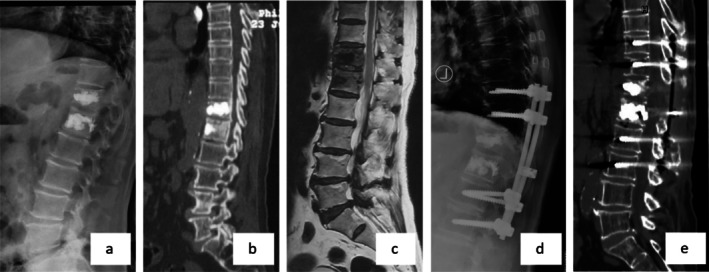
A 66‐year‐old female patient diagnosed with T12–L1 vertebral tuberculosis after T12–L1 vertebral augmentation. (a–c) Preoperative X‐ray, CT and MRI showed that there was bone destruction in the T12–L1 vertebrae and intervertebral space; the bone cement filled the vertebral body well, with good stability, and that no bone cement protruded into the spinal canal to compress the spinal cord. After 2 weeks of standard antituberculosis drug treatment, posterior thoracic canal decompression, bone graft fusion and internal fixation were performed; (d and e) Reexamination X‐ray and CT 18 months after the operation showed that the internal fixation was in good position without obvious loosening, no further bone destruction was found in the T12–L1 vertebrae compared with the preoperative period, and the lesion healed well.

**FIGURE 3:**
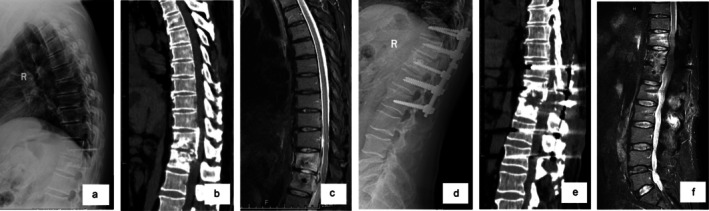
A 65‐year‐old male patient diagnosed with T11‐12 vertebral tuberculosis after T11‐12 vertebral augmentation. (a–c) Preoperative X‐ray, CT, and MRI revealed bone destruction in the T11‐12 vertebrae and intervertebral space; the bone cement filled the vertebral body well, with partial cement leakage into the spinal canal. After 2 weeks of standard antituberculosis drug treatment, posterior thoracic canal decompression, posterolateral bone graft fusion, and internal fixation were performed; (d and f) Postoperative 18‐month follow‐up X‐ray, CT, and MRI demonstrated well‐positioned internal fixation without obvious loosening. No progressive bone destruction was observed in the T11‐12 vertebrae compared with the preoperative period, and the focal lesion achieved favorable healing.

Within‐group comparisons revealed that in the conservative group, the VAS score was significantly lower at the last follow‐up than before treatment, and pain symptoms significantly improved $\left(z=3.771,p<0.001\right)$; in the surgical group, the VAS score was also significantly lower at the last follow‐up than before treatment, and the analgesic effect was significant (z=5.273,p<0.001). In the surgical group, CT at 5–9 months after the operation revealed good posterolateral bone graft fusion (Table 2). All surgical incisions healed by first intention, without adverse events such as incision infection, sinus formation, loosening, and rod or screw malfunctions (breaks) in internal fixators. At the last follow‐up, all the lesions in the conservative group had healed well. Imaging data for typical patients are shown in Figures 2 and 3.

**TABLE 2:** Comparison of VAS scores between the two groups.

Groups	Before treatment	Final follow‐up (18 months after treatment)	*z*	*p*
Surgical group	8.000 (7.000, 8.000)	2.000 (2.000, 3.000)	5.273	*p* < 0.001
Conservative group	7.500 (7.000, 8.000)	2.000 (2.000, 3.000)	3.771	*p* < 0.001
*z*	−1.887	−1.211		
*p*‐value	0.059	0.226		

### 3.3 Comparison of ESR

There was no significant difference in the ESR between the two groups before treatment (*p* > 0.05); the baseline ESR was comparable. The ESR in the surgical group was significantly greater than that in the conservative group on the 3rd day after treatment (*p* < 0.001), and the ESR in the surgical group was still greater than that in the conservative group at 3 months after treatment (*p* < 0.05). However, there was no significant difference in the ESR between the two groups at the last follow‐up (*p* > 0.05).

Within‐group comparisons revealed that the ESR in both groups at 3 months after treatment and at the last follow‐up was significantly lower than that before treatment and on the 3rd day after treatment (*p* < 0.05); the ESR in the surgical group was even lower at the last follow‐up than at 3 months after treatment (*p* < 0.05) (Table 3).

**TABLE 3:** Comparison of the ESR at different time points.

Groups	Before treatment	Postoperative day 3	3 months after treatment	Final follow‐up (18 months after treatment)	*χ* ^2^	*p*
Surgical group	49.000 (43.000, 54.000)	62.000 (56.000, 78.000)	14.000 (13.000, 15.000)^a^, ^b^	10.000 (9.000, 12.000)^a^, ^b^, ^c^	98.415	*p* < 0.001
Conservative group	47.500 (39.500, 57.000)	34.500 (24.500, 44.000)	13.000 (13.000, 14.000)^a^, ^b^	10.000 (9.000, 11.000)^a^, ^b^	48.326	*p* < 0.001
*z*	−0.367	−5.176	−2.355	−0.067		
*p*‐value	0.714	< 0.001	0.019	0.947		

^a^ *p* < 0.05 versus before treatment.

^b^ *p* < 0.05 versus postoperative day 3.

^c^ *p* < 0.05 versus 3 months after treatment.

### 3.4 Comparison of CRP Levels Between the Two Groups

Between‐group comparisons revealed that before treatment, there was no significant difference in the CRP level between the conservative group and surgical group $\left(z=-1.390,p=0.164\right)$; on the 3rd day after treatment, the CRP level was significantly greater in the surgical group than in the conservative group (z=−4.771,p<0.001). However, at 3 months after treatment and the last follow‐up, there were no significant differences in the CRP level between the two groups (*p* > 0.05).

Within‐group comparisons revealed that the overall time effect in both groups was statistically significant (*χ* ^2^ = 48.133 in the conservative group; *χ* ^2^ = 98.486 in the surgical group; both *p* < 0.001). Pairwise comparisons within groups revealed that the CRP levels in both groups at 3 months after treatment and at the last follow‐up were significantly lower than those before treatment and on the 3rd day after treatment (*p* < 0.05); the CRP level in the surgical group was even lower at the last follow‐up than at 3 months after treatment (*p* < 0.05) (Table 4).

**TABLE 4:** Comparison of CRP levels at different time points.

Groups	Before treatment	Postoperative day 3	3 months after treatment	Final follow‐up (18 months after treatment)	*χ* ^2^	*p*
Surgical group	33.670 (25.000, 40.000)	47.890 (39.210, 65.050)	3.170 (2.980, 3.270)^a^, ^b^	2.780 (2.560, 2.980)^a^, ^b^, ^c^	98.486	*p* < 0.001
Conservative group	40.280 (27.767, 47.250)	25.570 (21.068, 32.858)	3.190 (2.522, 3.290)^a^, ^b^	2.835 (2.365, 3.052)^a^, ^b^	48.133	*p* < 0.001
*z*	−1.39	−4.771	−0.244	−0.113		
*p*‐value	0.164	< 0.001	0.807	0.91		

^a^ *p* < 0.05 versus before treatment.

^b^ *P* < 0.05 versus postoperative day 3.

^c^ *p* < 0.05 versus 3 months after treatment.

### 3.5 Comparison of Daily Living Ability (MBI) Between the Two Groups

The MBI was used to evaluate the daily living ability of patients in the two groups. Between‐group comparisons revealed that before treatment, there was no significant difference in the MBI score between the conservative group and surgical group (z=−0.148,p=0.882); the baseline data were balanced and comparable. At 3 months after treatment, the MBI score for the surgical group was significantly greater than that for the conservative group (z=−5.305,p<0.001). However, at the last follow‐up, the MBI scores for the two groups were similar (z=−0.626,p=0.531).

Within‐group comparisons revealed that the overall time effect of MBI scores in both groups was statistically significant (*χ* ^2^ = 36.000 in the conservative group; *χ* ^2^ = 69.511 in the surgical group; both *p* < 0.001). Pairwise comparisons within groups revealed that in both groups, MBI scores at 3 months after treatment and at the last follow‐up were significantly higher than those before treatment; additionally, compared with those at 3 months after treatment, MBI scores at the last follow‐up were higher (*p* < 0.05) (Table 5).

**TABLE 5:** Comparison of MBI scores before treatment, 3 months after treatment and at the last follow‐up.

Groups	Before treatment	3 months after treatment	Final follow‐up (18 months after treatment)	*χ* ^2^	*p*
Surgical group	50.000 (45.000, 50.000)	85.000 (80.000, 90.000)^a^	95.000 (90.000, 95.000)^a^, ^b^	69.511	*p* < 0.001
Conservative group	45.000 (45.000, 50.000)	75.000 (70.000, 80.000)^a^	95.000 (90.000, 95.000)^a^, ^b^	36	*p* < 0.001
*z*	−0.148	−5.305	−0.626		
*p‐*value	0.882	< 0.001	0.531		

^a^ *p* < 0.05 versus before treatment.

^b^ *p* < 0.05 versus 3 months after treatment.

### 3.6 Improvement in Neurological Function

All patients were evaluated via the ASIA neurological classification before treatment; 7 patients were grade B, 15 patients were grade C, 18 patients were grade D, and 13 patients were grade E. At the last follow‐up, 2 patients were grade C, 14 patients were grade D and 37 patients were grade E. Between‐group comparisons revealed that before treatment, the ASIA classification of patients in the conservative group and surgical group was similar, with no statistically significant difference (z=−0.150,p=0.881); the ASIA classification at baseline was balanced and comparable. At the last follow‐up, there was still no statistically significant difference in ASIA classification between the two groups (z=−0.387,p=0.699).

Within‐group comparisons revealed that the ASIA classification in the conservative group significantly improved from before treatment to the final follow‐up (z=3.272,p=0.001). In the surgical group, the ASIA classification also significantly improved from before treatment to the last follow‐up, indicating that neurological function significantly improved (z=4.194,p<0.001) (Table 6).

**TABLE 6:** Comparison of ASIA classification at different time points between the two groups.

Groups	Before treatment	Final follow‐up (18 months after treatment)	*z*	*p*
Surgical group	2.000 (2.000, 3.000)	1.000 (1.000, 2.000)	4.194	*p* < 0.001
Conservative group	2.000 (2.000, 3.000)	1.000 (1.000, 2.000)	3.272	0.001
*z*	−0.15	−0.387		
*p‐*value	0.881	0.699		

## 4 Discussion

Early diagnosis of spinal tuberculosis remains challenging. Elderly patients often present with nonspecific clinical and imaging manifestations, leading to frequent misdiagnoses of vertebral compression fractures and inappropriate vertebral augmentation. Notably, spinal tuberculosis is an absolute contraindication to vertebral augmentation. The presence of bone cement and delayed antituberculosis treatment often exacerbate infection, accelerate disease progression, and may lead to catastrophic neurological consequences. Currently, there is no definite conclusion on managing the misdiagnosis of spinal tuberculosis followed by vertebral augmentation.

The retrospective cohort study shows that compared with conservative treatment, surgical treatment results in faster symptomatic and functional recovery in patients with neurological compression after mismanaged vertebral augmentation for spinal tuberculosis. For patients without significant neurological compression or those who are unfit for surgery, conservative treatment achieves satisfactory long‐term efficacy, although the recovery time is longer. Long‐term outcomes are comparable between the two strategies.

### 4.1 Clinical Characteristics of Patients With Misdiagnosed Spinal Tuberculosis Who Underwent Vertebral Augmentation

With the aging of the population in China, osteoporosis has become a prominent public health problem. As a common complication of osteoporosis, osteoporotic vertebral fracture has become the preferred clinical surgical treatment because of its advantages of satisfactory analgesic effects and early ambulation. Polymethyl methacrylate (PMMA) bone cement is widely used in elderly individuals [17]. However, the early symptoms of elderly individuals with spinal tuberculosis are insidious, and the clinical manifestations overlap strongly with those of osteoporotic vertebral fractures, both of which manifest as low back pain centered on the diseased vertebrae. Some patients may have vertebral compression fractures due to trauma, which are prone to misdiagnosis and mistreatment [18, 19, 20]. After misdiagnosis, patients undergo vertebral augmentation, which delays tuberculosis treatment and may aggravate the disease. The main clinical symptoms are no obvious relief of pain or aggravation of chest or low back pain and limited mobility shortly after vertebral augmentation.

On the basis of the 53 patients in this study cohort (mean disease course of 4.2 months, rapid disease progression) and patients in related studies, misdiagnosis is caused by the synergistic effect of multiple factors.

1. Interference from patients' own physiological characteristics and underlying diseases: Most of the patients in this cohort were elderly patients with decreased immune function. After infection with *M. tuberculosis* , typical toxicity symptoms such as night sweats and low‐grade fever in the afternoon were insidious and manifested only as nonspecific symptoms such as fatigue and poor appetite; in addition, the patients' cognitive and expressive ability was poor, medical history collection was incomplete, and underlying diseases and insufficient nutrition further weakened the body's anti‐tuberculosis ability, aggravated the insidious nature of the disease, increased the probability of misdiagnosis, and accelerated disease progression.
2. Oversight in the diagnosis and treatment process: It seems as though doctors rely too much on initial imaging findings, ignoring key differential factors such as the ESR, CRP level, and T‐SPOT results [21] before surgery (52 patients in this cohort had elevated ESRs and CRP levels, and 36 patients had positive T‐SPOT results), only confirming the diagnosis of vertebral wedge‐shaped deformation without ruling out the possibility of pathological fractures. Moreover, for the cohort in this study, no lesion tissue was taken for pathological examination during vertebral augmentation, resulting in delayed antituberculosis treatment, which is one of the core reasons for rapid disease progression in this group, consistent with the conclusions of related studies. The lack of examination equipment in primary medical institutions further aggravates diagnosis oversight.
3. Overlapping imaging manifestations and insufficient early specificity of diseases: The imaging manifestations of elderly individuals with spinal tuberculosis and osteoporotic vertebral fractures overlap greatly, which is an important objective factor for misdiagnosis [18]. The latter mostly occurs in T11–L2, with single vertebral wedge‐shaped deformation and anterior–middle column compression as typical manifestations, whereas early spinal tuberculosis in elderly individuals manifests only as mild vertebral compression and wedge‐shaped deformation, without obvious erosive bone destruction, intervertebral space stenosis or paravertebral abscess shadow [22], making it difficult to distinguish from osteoporotic vertebral fractures.
4. Limited understanding that bone cement aggravates the consequences of misdiagnosis: Although the commonly used bone cement PMMA can relieve pain quickly and promote early ambulation, it can aggravate disease progression when used in patients with spinal tuberculosis. On the one hand, the sclerotic structure formed by bone cement in the diseased vertebrae hinders the diffusion of antituberculosis drugs and reduces the therapeutic effect; on the other hand, the unstable bone cement mass is similar to “sequestrum,” which aggravates the local immune response, promotes the proliferation of granulation tissue and caseous necrotic tissue, compresses the spinal cord and nerve roots, and leads to the rapid progression of paraplegia symptoms.

### 4.2 Key Points of Differential Diagnosis

The following key points should be considered in the differential diagnosis of elderly individuals with spinal tuberculosis, osteoporotic vertebral fractures and spinal tumors. ① There should be an emphasis on laboratory examinations. In this group, 52 patients (52/53) had elevated ESRs and CRP levels, and 36 patients had positive T‐SPOT results. The combined detection of the above three indicators can significantly improve the diagnostic accuracy for elderly individuals and atypical imaging cases. For elderly patients with chest and low back pain, such examinations should be performed routinely before surgery to avoid misdiagnosis caused by oversight. ② Differential imaging features should be considered. Osteoporotic vertebral fractures are mostly single vertebral wedge‐shaped deformations, without erosive bone destruction or intervertebral space stenosis, and paravertebral soft tissue shadows are limited; spinal tuberculosis can present as vertebral wedge‐shaped deformation; and, in the later stage, anterior–middle column destruction of the vertebral body, intervertebral disc involvement, intervertebral space stenosis and fusiform paravertebral soft tissue swelling shadows occur. In this study, MRI revealed 37 cases (69.81%) complicated with paravertebral abscess. ③ A gold standard for pathological diagnosis should be established. For patients with atypical imaging manifestations and laboratory examinations suggesting infection, a clear diagnosis can be made by preoperative puncture biopsy or intraoperative pathological examination.

### 4.3 Treatment Measures and Surgical Indications

On the basis of the results of the above analysis, the core problem of spinal tuberculosis being misdiagnosed and patients undergoing vertebral augmentation is delayed early diagnosis. Therefore, standardizing the diagnosis process and strengthening the awareness of differential diagnosis are keys to avoiding such misdiagnosis [9, 23]. Standardized antituberculosis chemotherapy is key for treating spinal tuberculosis. For patients who are misdiagnosed and undergo vertebral augmentation, the timely initiation of effective antituberculosis drug treatment is the basis for controlling disease progression and improving prognosis [24].

In this cohort, owing to misdiagnosis and delayed antituberculosis treatment, the disease progressed. In both the surgical group and the conservative group, satisfactory long‐term efficacy was achieved (improved VAS score, MBI score and ASIA classification) after 18 months of standard antituberculosis treatment was completed. In the short term, the surgical group had more advantages in terms of the recovery of daily functional ability; however, transient increases in the ESR and CRP levels were observed. For drug‐resistant patients, the regimen should be adjusted according to drug susceptibility test results, and the course of treatment should be extended to 18–24 months.

Treatment plans should be formulated individually [25, 26], comprehensively considering the condition of bone cement mass, degree of neurological function injury, patient age and surgical tolerance. Most patients with spinal tuberculosis are elderly and have multiple medical complications and high surgical risk; thus, surgical indications should be strictly controlled: ① progressive nerve function deterioration due to compression of the sequestrum, abscess and unstable bone cement mass; ② severe spinal instability or deformity; ③ no improvement or even progression of the disease after standardized antituberculosis treatment; and ④ risk of loosening and displacement of an unstable bone cement mass [27, 28].

Conservative treatment is suitable for patients who lack obvious nerve compression, who have stable bone cement, who are intolerant to surgery, or who have good anti‐tuberculosis treatment effects. For patients with obvious spinal cord and nerve compression but who refuse surgery, conservative treatment can also be selected [29, 30]. The core is standardized antituberculosis treatment, supplemented by nutritional support, symptomatic treatment, and basic disease control [31].

### 4.4 Limitations and Strengths

Several strengths of this study should be noted. ① This study targeted a special and clinically intractable cohort of spinal tuberculosis patients with inappropriate vertebral augmentation. Relevant high‐quality clinical studies remain scarce, and our results supplement available clinical evidence for the management of such complicated cases. ② All enrolled patients were screened with unified inclusion and exclusion criteria and received long‐term follow‐up of 18–36 months. Multiple clinical indicators including ESR, CRP level, VAS score, ASIA grade, and MBI score were comprehensively evaluated, which ensures the integrity and persuasiveness of the outcome analysis.

This study still has limitations. ① This study involved a retrospective analysis, with data from a single center, a relatively small sample size, and a limited follow‐up time; therefore, certain biases may have been introduced, which could affect the robustness of the conclusions.② Treatment grouping may also have introduced additional bias. ③Confounding factors (e.g., surgical tolerance, comorbidities, severity of pathology) were not included in the multivariate analysis. In future studies, we will carry out larger sample prospective, multicenter studies to verify the conclusions of the present study.

## 5 Conclusion

In conclusion, the misdiagnosis of spinal tuberculosis followed by vertebral augmentation results from the synergistic effects of multiple factors, such as patient characteristics, diagnosis process oversight, imaging overlap, and a limited understanding of bone cement, which are closely related to medical resources and physician level. Clinically, it is necessary to strengthen the awareness of the differential diagnosis of elderly patients with spinal tuberculosis, standardize preoperative examinations, combine laboratory examinations, multimodal imaging, and pathological examinations, comprehensively rule out the possibility of pathological fracture, and reduce the misdiagnosis rate. For patients with misdiagnosis, standardized antituberculosis treatment should be initiated in a timely manner, individualized plans should be formulated according to the specific condition of each patient, and prevention strategies should be developed to improve diagnosis and treatment quality, reduce misdiagnosis and mistreatment, and improve long‐term disease prognosis.

## Author Contributions

**Yatao Wang:** conceptualization, investigation, methodology, validation, writing – original draft. **Enning Cui:** investigation. **Long Yu:** writing – review and editing, supervision. **Zhanpeng Luo:** investigation, formal analysis. **Cunshuo Wang:** investigation. **Xu Cui:** visualization, writing – review and editing, supervision. **Yunfeng Wu:** investigation. **Bo Wang:** investigation. **Ning Liu:** investigation. **Xiong Xu:** conceptualization, methodology, validation, writing – original draft, investigation. **Litao Li:** investigation.

## Funding

The authors have nothing to report.

## Conflicts of Interest

The authors declare no conflicts of interest.

## Data Availability

Anonymized participant data for this study will be available upon request to the corresponding author.
